# Predictors of mortality among adolescents and young adults living with HIV on antiretroviral therapy in Dar es Salaam, Tanzania: a retrospective cohort study

**DOI:** 10.1002/jia2.25886

**Published:** 2022-02-22

**Authors:** Maryam A. Amour, Grace A. Shayo, Mecky M. Matee, Lameck Machumi, Angelica Rugarabamu, Eric A. Aris, Bruno F. Sunguya, Ferdinand M. Mugusi

**Affiliations:** ^1^ Department of Community Health Muhimbili University of Health and Allied Sciences Dar es Salaam Tanzania; ^2^ Department of Internal Medicine Muhimbili University of Health and Allied Sciences Dar es Salaam Tanzania; ^3^ Department of Microbiology and Immunology Muhimbili University of Health and Allied Sciences Dar es Salaam Tanzania; ^4^ Management and Development for Health Dar es Salaam Tanzania

**Keywords:** acquired immunodeficiency syndrome, adolescent, HIV, mortality, Tanzania, young adult

## Abstract

**Introduction:**

Global AIDS‐related deaths have declined by only 10% among adolescents since its peak in 2003. This is disproportionately low compared to a decline of 74% among children aged 0–9 years old. We determined the magnitude of, and predictors of mortality among adolescents and young adults living with HIV on antiretroviral therapy (ART) in Dar‐es‐Salaam, Tanzania.

**Methods:**

A retrospective cohort study was conducted among adolescents (aged 10–19) and young adults (aged 20–24) living with HIV and enrolled in care and treatment centres in Dar es Salaam, Tanzania between January 2015 and December 2019. Data were analysed using STATA version 16. Cumulative hazard curves were used to estimate and illustrate 1‐year mortality. Predictors for mortality were assessed by the Fine and Gray competing risk regression model. Sub‐hazard ratios (SHR) and 95% confidence intervals (95% CI) were then reported.

**Results:**

A total of 15,874 young people living with HIV were included: 4916 (31.3%) were adolescents and 10,913 (68.7%) were young adults. A total of 3843 (77.5%) adolescents and 9517 (87.2%) young adults were female. Deaths occurred in 2.3% (114/4961) of adolescents and 1.2% (135/10,913) of young adults (*p* < 0.001). Over a follow‐up of 9292 person‐years, the mortality rate was 3.8 per 100 person years [95% CI 3.2–4.6/100 person‐years] among adolescents and 2.1 per 100 person‐years among young adults [95% CI 1.8–2.5/100 person‐years]. Independent predictors of mortality among adolescents were male sex (adjusted (SHR) aSHR = 1.90, 95% CI: 1.3–2.8), CD4 count < 200 cells/mm^3^ (aSHR = 2.7, 95% CI: 1.4–5.0) and attending a private health facility (aSHR = 1.7, 95% CI: 1.1–2.5). Predictors of mortality among young adults were CD4 count < 200 cells/mm^3^ (aSHR = 2.8, 95% CI 1.7–4.5), being underweight (aSHR = 2.1, 95% CI: 1.4–3.3) and using nevirapine‐based therapy (aHR = 8.3, 95% CI: 3.5–19.5).

**Conclusions:**

The mortality rate for persons living with HIV and on ART in Tanzania was significantly higher in adolescents than young adults. Age‐ and sex‐specific risk factors identify targets for intervention to reduce mortality among affected adolescents and young adults.

## 1 INTRODUCTION

The introduction of antiretroviral therapy (ART) has resulted into a global decline in AIDS‐related deaths [1]. However, AIDS remains the second leading cause of death among adolescents worldwide [1]. In 2020, an estimated, 680,000 people died of HIV‐related illnesses [2] with 32,000 of these among adolescents aged 10–19 years [3].

Globally, since its peak in 2003, the number of AIDS‐related deaths has declined by 64% in all age groups and by 74% among children 0–9 years [4, 5]. In contrast, the reduction in AIDS‐related mortality among adolescents has only been 10% [4].

Data on AIDS‐related mortality in Tanzania have been limited to studies in younger children and adults; data have not been reported on adolescents [6]. Adolescent populations have unique features, which influence access and utilization of HIV‐related care and treatment services [7], and, therefore, warrant special consideration.

In 2019, about 1.4 million people (5%) were living with HIV in Tanzania, with 27,000 reported AIDS‐related deaths. With scaling up of ART access in the past decade, the national number of newly acquired HIV infections and deaths has declined by 13% and 50%, respectively [8]. The current prevalence of HIV infection is estimated to be 1.3% and 0.7% among adolescent girls and boys aged 10–19, respectively [8, 9], while the prevalence in young adults aged 20–24 is 2.2% [8].

Published data indicate that some adolescents and young adults living with HIV may die within the first year of ART initiation [10, 11, 12, 13]. The predictors of mortality among adolescents and young adults living with HIV are not uniform across studies [7, 14, 15, 16]. Identified predictors include low CD4 count, advanced WHO Stage clinical disease, short duration of ART, sex and age [7, 14, 15, 16].

In view of the limited data on mortality among adolescents and young adults living with HIV in Tanzania and the diversity of the predictors of mortality, the present study was designed to determine mortality rates and predictors of mortality among adolescents and young adults living with HIV within 1 year of ART initiation.

## 2 METHODS

### 2.1 Study design and setting

This retrospective cohort study was conducted in care and treatment clinics (CTCs) in Dar‐es‐Salaam, which are supported by the Management and Development for Health (MDH) organization. MDH is a Tanzania‐based non‐governmental organization funded by the President's Emergency Plan for AIDS Relief (PEPFAR) through Centers for Disease Control and Prevention to provide ART access and HIV management and care in Tanzania [17]. MDH provides infrastructure, laboratory and technical support to these clinics. Dar‐es‐Salaam is the largest city as well as main commercial city in Tanzania, with an approximate population of 6 million individuals [18]. The overall prevalence of HIV in Dar es Salaam is 4.3%, which is close to the national average of 4.9% [8].

### 2.2 Study population and duration

This study included all adolescents aged 10–19 years and young adults aged 20–24 years with confirmed HIV infection who had initiated ART at enrolment. Data were censored whenever any of the following appeared first: (1) death, (2) loss to follow up, (3) turning 19 years and 11 months of age for adolescents, or (4) turning 24 years and 11 months of age for young adults and (5) 1 year after ART initiation. Loss to follow up was defined as patients not attending care for 3 months. The study covered a period of 5 years from 1st January 2015 to 31st December 2019. Each participant was retrospectively followed up for 1 year from the date of ART initiation. The main outcome was mortality within 1 year of ART initiation.

### 2.3 Sampling and sample size estimation

A total of 22,690 individuals with available HIV data were studied. Of these, 30% (6816) were excluded because of missing information on ART, stopped ART or were transfer to an ART centre not supported by MDH. The remaining 15,874 individuals were included in the final analysis. A minimum sample of 169 individuals in each group was needed to have 90% power at a significance level of 0.05 for two‐sample comparison of survivor functions.

### 2.4 Study measures and data extraction

We extracted de‐identified demographic and clinical data from the CTC‐2 electronic based records used throughout the MDH‐supported CTC clinics in Dar‐es‐Salaam. Baseline data were collected on the first day of attendance at the CTC clinic, including demographic information and laboratory tests. Demographic data collected included sex and private versus public treatment centre. Clinical data included ART regimen combination at baseline (dolutegravir, nevirapine and efavirenz or protease inhibitor‐based), virological suppression (yes or no), WHO Stages (I, II, III and IV), absolute CD4 count, body weight and height and mortality (yes or no). Body mass index (BMI) was computed by age‐ and sex‐adjusted Z‐scores using the AnthroPlus WHO child growth standard software [19].

### 2.5 Data processing and analysis

The data were analysed using STATA version 16. Baseline characteristics of the study population were summarized with descriptive statistics using proportions for categorical variables and medians with interquartile ranges (IQR) for continuous variables. Cumulative incidence curves for mortality in the presence of competing risks were presented by age and sex. A *p*‐value of less than 0.05 was considered statistically significant for both median and proportions.

We assessed the initial predictors for mortality in a univariate analysis using Fine and Gray's competing risk regression analysis [20]. In this analysis, loss to follow up was considered a competing event. The resulting sub‐hazard ratio (SHR) was interpreted in a similar way to the hazard ratio, taking into account the hazard of competing events [20, 21]. We then utilized Fine and Gray's competing risk regression model to assess the predictors of death within 1 year of ART initiation [20]. Variables were included in the multivariate model if they had a *p* < 0.2 in univariate analysis. We selected the final model using a backward elimination procedure and retained all variables in the model that had a *p* < 0.05. The adjusted sub‐hazard hazard ratios (aSHR) were reported with their 95% confidence intervals (95% CI). This analysis assessed sex, CD4 count, WHO Stage, facility type, ART regimen, TB co‐infection and BMI as covariates. Viral load data were not included in the regression analysis because the majority of those eligible for viral load measurement did not have the measurements. Additionally, missing viral load values might have been associated with the mortality observed.

We used multiple imputation with chained equation to handle the remaining missing data. In the imputation, we used ordinal logistic regression for the categorical CD4 count, WHO Staging categories and BMI categories controlling for age, sex and other baseline covariates. In this analysis, 10 imputations were conducted [22].

### 2.6 Ethical approval

Ethical clearance was obtained from the Research and Publications Committee of the Muhimbili University of Health and Allied Sciences with Ref.No.DA.282/298/01. Permission to use these data was obtained from the National AIDS Control Program Tanzania through the Ministry of Health Gender Elderly and Children.

## 3 RESULTS

Between 2015 and 2019, a total of 4961 (31.3%) adolescents aged 10–19 and 10,913 (68.7%) young adults aged 20–24 living with HIV (totalling 15,874) were enrolled to initiate ART at MDH‐supported CTC clinics in Dar es Salaam (Table 1). The majority in both age groups were female: 77.5% (3243/4961) among adolescents and 87.2% (9517/10,913) among young adults. Most attended government facilities for care, totalling 4037 (77.6%) among adolescents and 9122 (79.6%) among young adults. More young adults were enrolled at WHO Stage I (*n* = 8104, 74.4%) compared with adolescents (*n* = 3025, 61.1%). Twice as many adolescents (1.4%) were enrolled with WHO Stage IV compared to young adults (0.7 %). Virological suppression was observed in 33.6% of adolescents and 34.1% of young adults. Most of the adolescents and young adults had normal BMIs (59.4% and 62.2%, respectively). Less than 1% in both groups had TB co‐infection at the initiation of ART. The number of adolescents and young adults enrolled in CTC clinics and on ART increased more than two‐fold from 2015 to 2019.

**Table 1:** Baseline socio‐demographic and clinical characteristics of adolescents and young adults enrolled in HIV care and treatment clinics

Characteristics	Adolescents	Young adults
Total *N* = 15,874	*N* = 4961	*N* = 10,913
Age (median, IQR)	18.1 [15.5–19.2]	22.2 [21.1–23.2]
Sex, number (%)		
Male	1118 (22.5)	1396 (12.8)
Female	3843 (77.5)	9517 (87.2)
Facility type, number (%)		
Public	4037 (77.6)	9122 (79.6)
Private	924 (22.4)	1791 (20.4)
Regimen combination, number (%)		
Dolutegravir based	201 (4.1)	559 (5.1)
Efavirenz based	4182 (84.3)	10,274 (94.1)
Nevirapine based	524 (10.6)	69 (0.6)
Protease inhibitor based	45 (0.9)	3 (0.03)
Others	9 (0.2)	8 (0.1)
WHO Stage, number (%)		
WHO Stage 1	3025 (61.1)	8104 (74.4)
WHO Stage 2	1028 (20.8)	1605 (14.8)
WHO Stage 3	827 (16.7)	1101 (10.1)
WHO Stage 4	68 (1.4)	78 (0.7)
Missing	13 (0.3)	25 (0.2)
CD4 count in cells/mm^3^, number (%)		
< 200	340 (6.9)	541 (4.9)
200–349	360 (7.3)	845 (7.7)
350–500	429 (8.7)	951 (8.7)
> 500	730 (14.7)	1646 (15.1)
Missing	3102 (62.5)	6930 (63.5)
Viral load at 6 months, number (%)		
Suppressed	1668 (33.6)	3716 (34.1)
Not suppressed	455 (9.2)	502 (4.6)
Missing	2838 (57.2)	6695 (61.4)
BMI (kg/m^2^) categories, number (%)		
BMI <18.5 (underweight)	833 (16.8)	882 (8.1)
18.5 ≤ BMI < 24 (normal)	2225 (44.9)	5684 (52.1)
24 ≤ BMI < 27 (overweight)	538 (10.8)	1873 (17.2)
BMI ≥ 27 (obese)	149 (3.0)	703 (6.4)
Missing	1216 (24.5)	1771 (16.2)
TB status at enrolment, number (%)		
TB co‐infection	40 (0.8)	64 (0.6)
No TB co‐infection	4921 (99.2)	10,849 (99.4)
Year started ART		
2015	466 (9.4)	957 (8.8)
2016	742 (15.0)	1444 (13.2)
2017	1493 (30.1)	2905 (26.6)
2018	1184 (23.9)	2660 (24.4)
2019	1076 (21.7)	2947 (27.0)

Abbreviations: BMI, body mass index; IQR, interquartile range; TB, tuberculosis; WHO, World Health Organization.

There were significantly more deaths within 1 year of ART initiation among adolescents [2.3% (114/4961)] compared to young adults [1.2% (135/10,913)], *p*‐value < 0.001. The overall mortality rate per year for both adolescents and young adults over a total follow‐up period of 9292 person‐years was 2.7 per 100 person‐years [95% CI: 2.4–3.0]. The mortality rate over 3005 person‐years of follow up among adolescents was 3.8 per 100 person‐years [95% CI 3.2–4.6/100 person years]. The mortality rate over 6287 person‐years of follow up among young adults was 2.1 per 100 person‐years [95% CI 1.8–2.5/100 person‐years]. The adolescents were 80% more likely to die within 1 year of ART initiation compared to young adults with an incidence risk ratio 1.8 [95% CI 1.4–2.3/100] (Table S1).

**Figure 1:**
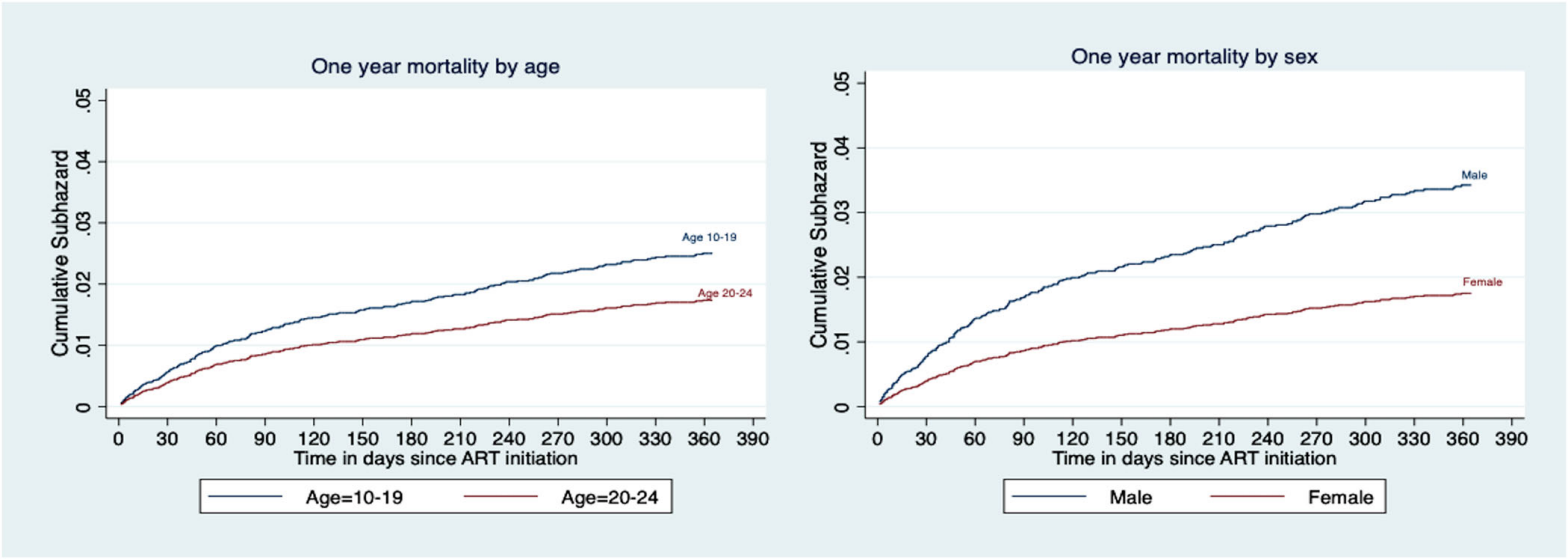
Cumulative subhazard curves showing 1‐year mortality by age and sex among adolescents and young adults living with HIV and on ART for 1 year.

The cumulative incidence of mortality with loss to follow up as a competing risk within the first year of ART initiation is shown in Figure 1. Compared to young adults, adolescents had a significantly higher probability of dying in the first year of ART initiation, 1.8% versus 2.5%, respectively; *p* < 0.001. The cumulative 1‐year mortality was significantly higher in males compared to females (3.4% vs. 1.8%, respectively) *p* < 0.001. In both groups, the mortality was highest within the first 90 days.

The predictors of mortality among adolescents aged 10–19 years old are summarized in Table 2. In univariate analysis, the predictors of 1‐year mortality following enrolment among adolescents were male sex, being underweight, WHO Stages II, III or IV, CD4 count < 200 cells/mm^3^, nevirapine or protease inhibitor (PI)‐based therapy and attending a private health facility. In multivariate analysis, we adjusted for sex, CD4 count, regimen combination and facility type. Compared to female adolescents, the risk of dying was 90% higher among male adolescents (aSHR = 1.9, 95% CI: 1.3–2.8). Compared to adolescents with CD4 counts > 500 cells/mm^3^, the risk of dying was three‐fold higher with a CD4 count < 200 cells/mm^3^ (aSHR = 2.7, 95% CI: 1.4–5.0). The risk of dying was 70% higher among adolescents who received care from a private health facility (aSHR = 1.7, 95% CI: 1.1–2.5). The ART regimen was not significantly associated with increased risk of mortality after adjusting for other factors (Table 2).

**Table 2:** Competing risk regression analysis for the predictors of mortality among adolescents aged 10–19 years old living with HIV

	Unadjusted		Adjusted	
Variable	SHR (95% CI)	*p*‐value	aSHR (95% CI)	*p*‐value
Sex				
Female	1.0		1.0	
Male	2.4 (1.7–3.6)	< 0.001	1.9 (1.3–2.8)	0.001
BMI				
Normal	1.0			
Underweight	1.7 (1.1–2.8)	0.036		
Overweight	0.8 (0.3–1.7)	0.515		
CD4 count (cells/mm^3^)				
> 500	1.0		1.0	
350–500	1.5 (0.7–3.1)	0.311	1.4 (0.7–3.1)	0.336
200–349	1.6 (0.7–3.5)	0.278	1.4 (0.6–3.3)	0.373
< 200	3.1 (1.7–5.8)	< 0.001	2.7 (1.4–5.0)	0.003
WHO Stage				
WHO Stage I	1.0			
WHO Stage II	2.0 (1.2–3.4)	0.011		
WHO Stage III	5.2 (3.3–8.1)	< 0.001		
WHO Stage IV	15.2 (7.4–29.7)	< 0.001		
Regimen combination				
EFV based	1.0		1.0	
DTG based	1.9 (0.8–4.8)	0.158	1.8 (0.7–4.4)	0.199
NVP based	2.0 (1.3–3.2)	0.002	1.5 (0.9–2.4)	0.093
PI based	3.5 (1.1–10.9)	0.031	2.2 (0.7–6.7)	0.150
TB co‐infection				
No TB	1.0	0.967		
Yes	0.9 (0.1–7)			
Facility type				
Public	1.0		1.0	
Private	1.8 (1.2–2.6)	0.004	1.7 (1.1–2.5)	0.013

Note: Global *p*‐value <0.001. Adjusted for sex, CD4 count regimen combination and facility type.

Abbreviations: aSHR, adjusted subhazard ratio; BMI, body mass index; CI, confidence interval; DTG, dolutegravir; EFV, efavirenz; NVP, nevirapine; PI, protease inhibitor; SHR, subhazard ratio; TB, tuberculosis; WHO, World Health Organization.

Predictors of mortality among young adults aged 20–24 are summarized in Table 3. In univariate analysis, the predictors of 1‐year mortality following enrolment among young adults were male sex, being underweight, WHO Stages III and IV, CD4 count < 200 cells/mm^3^ and use of nevirapine. Being overweight was associated with reduced risk of 1‐year mortality. In multivariate analysis after adjusting for sex, BMI, CD4 count and regimen combination, the risk of dying among young adults was two‐fold higher among those who were underweight (aSHR = 2.1, 95% CI: 1.4–3.3), three‐fold higher when the CD4 count < 200 cells/mm^3^ (aSHR = 2.8, 95% CI: 1.7–4.5) and eight‐fold higher among those on nevirapine‐based therapy (aSHR = 8.3, 95% CI: 3.5–19.5), *p* < 0.001 but 50% lower in overweight young adults (aSHR = 0.5, 95% CI: 0.2–0.9) (Table 3).

**Table 3:** Competing risk regression analysis for the predictors of mortality among young adults aged 20–24 years old living with HIV

	Unadjusted		Adjusted	
Variable	HR (95% CI)	*p*‐value	aHR (95% CI)	*p*‐value
Sex				
Female	1.0		1.0	
Male	2.0 (1.3–2.8)	0.002	1.4 (0.9–2.2)	0.108
BMI				
BMI <18.5 (underweight)	1.0		1.0	
18.5 ≤ BMI < 24 (normal)	2.4 (1.6–3.7)	< 0.001	2.1 (1.3–3.3)	0.001
24 ≤ BMI < 27 (overweight)	0.4 (0.2–0.9)	0.036	0.5 (0.2–0.9)	0.044
BMI ≥ 27 (obese)	0.8 (0.3–1.8)	0.548	0.8 (0.4–2.1)	0.721
CD4 count (cells/mm^3^)				
> 500	1.0		1.0	
350–500	1.9 (0.6–2.3)	0.597	1.1 (0.6–2.1)	0.719
200–349	1.5 (0.8–3.0)	0.235	1.4 (0.7–2.8)	0.326
< 200	3.2 (2.0–5.2)	< 0.001	2.8 (1.7–4.5)	< 0.001
WHO Stage				
WHO Stage I	1.0			
WHO Stage II	4.1 (2.6–6.4)	< 0.001		
WHO Stage III	8.0 (5.3–12.2)	< 0.001		
WHO Stage IV	30 (15.5–59.9)	< 0.001		
Regimen combination				
EFV based	1.0		1.0	
DTG based	1.8 (0.8–4.1)	0.164	1.7 (0.7–3.9)	0.208
NVP based	8.3 (3.6–19.1)	< 0.001	8.2 (3.5–19.6)	< 0.001
TB co‐infection				
No	1.0	0.208		
Yes	2.4 (0.6–9.8)			
Facility type				
Public	1.0			
Private	0.9 (0.6–1.4)	0.801		

Note: Global *p*‐value <0.001. Adjusted for sex, BMI, CD4 and regimen combination at enrolment.

Abbreviations: aSHR, adjusted subhazard ratio; BMI, body mass index; CI, confidence interval; DTG, dolutegravir; EFV, efavirenz; NVP, nevirapine; PI, protease inhibitor; SHR, subhazard ratio; TB, tuberculosis; WHO, World Health Organization.

## 4 DISCUSSION

This retrospective cohort study of adolescents and young adults living with HIV on ART found a two‐fold higher risk of mortality for adolescents compared to young adults. The overall mortality rate within the first year of ART initiation was significantly higher in adolescents compared to young adults, 3.8 per 100 among adolescents versus 2.1 per 100 person‐years among young adults. There are several factors that might explain the higher mortality observed among adolescents. Previous studies have attributed these differences to structural barriers, such as restrictive laws on the age of consent for self‐care, poor linkage to CTC care and poor adherence to ART [15, 23]. Despite the known need for protection from HIV infections and other reproductive health risks, their age and social and economic statuses limit the access of adolescents to information and services, which can lead to poor outcomes, including mortality [24].

In line with previous reports from Ethiopia and Zimbabwe, mortality was highest during the first 90 days after initiation of ART for both adolescents and young adults, with more deaths seen in the former [25, 26, 27]. The finding that the peak mortality among adolescents occurred within 90 days of HIV diagnosis raises the possibility that youth present late for care because of stigma and fear concerning HIV/AIDS, and/or may have poor access to testing services.

In keeping with other studies in sub‐saharan Africa (SSA), mortality in adolescent males was twice as high as in females [7, 16, 28, 29, 30, 31]. Published studies have shown that the reasons for increased mortality observed in men living with HIV are poor health‐seeking behaviour, delay in HIV status disclosure, poor access to HIV services, including testing and treatment, poor engagement to HIV services, late presentation to healthcare, delayed initiation of treatment, poor adherence to ART and poor retention [7, 28, 29, 30, 32]. According to the Tanzania HIV Indicator Survey of 2017, 79% of adolescent men had never been tested for HIV despite high levels of sexual activity, and treatment requires diagnosis [8].

We observed that low CD4 counts at baseline were associated with an increased risk of mortality within 1 year following ART initiation. This is in keeping with the findings of a global cohort collaborative study involving adolescents living with HIV in Asia Pacific, the Caribbean, Central and South America, and SSA that showed adolescents with CD4 < 200 cells/mm^3^ were more likely to die within a year of diagnosis [14]. Similar findings were observed among adolescents and young adults in several other studies [14, 15, 16, 26, 33]. The CD4 cell count at the initiation of ART is the most important predictor of disease progression, and determines opportunistic infection (OI) risk stratification, when to start or stop prophylaxis for OI and guidelines for monitoring response to treatment. CD4 count provides a direct measurement of a patient's immune status [34, 35]. It is well known that a CD4 count of less than 200 cells/mm^3^ is a critical threshold for heightened risk of death and similarly used as the current WHO definition of advanced HIV disease [35].

The present study showed that being underweight was a predictor of mortality in young adults, a finding that was also observed in a study in Ethiopia [33]. Being underweight at enrolment into treatment could serve as an indication of a severe disease as well as serve as poor prognostic indicator [36]. We, therefore, anticipated that underweight would be associated with reduced immunity and risk of co‐infections, such as tuberculosis, which might have contributed to mortality in this subgroup. However, other studies have shown that being underweight was not a predictor of mortality among youths or young adults living with HIV [31, 37, 38]. We found that being overweight was associated with a reduced risk of 1‐year mortality by 50% among young adults. This is in keeping with other studies that reported similar findings [39, 40, 41, 42]. Previous studies have associated overweight in individuals living with HIV with access to ART, virological suppression, higher CD4 counts, reduced risks of OI, healthier nutrition and higher levels of education, occupation and income, slower disease progression and decreased mortality [36, 42]. However, the protective advantage of being overweight in this population needs to be distinguished from the well‐known adverse effects of overweight and obesity in the general population [42].

Lastly, we observed that adolescents were less likely to die in public facilities, which could be due to the greater experience in standardized HIV care and treatment in public facilities before they were rolled out to involve private facilities. However, other studies involving adolescents and young adults showed that private or public facility type was not a predictor of 1‐year mortality [15, 16].

### 4.1 Strengths and limitations

This study has important limitations, which need to be considered while interpreting the findings. First, owing to the retrospective nature of data collection, we encountered missing data.

In this study, viral load data were not included in the regression analysis because the majority of those eligible for viral load measurement were missing viral load values. Further, we were not able to include important potential predictors of mortality, such as routes of HIV transmission, whether perinatally acquired or not. Since our focus was adolescents, this might have resulted in survivor bias because cases who were perinatally acquired would have to survive in order to have initiated ART when reaching adolescent age. In addition, due to the nature of the study, significant loss to follow‐up might have underestimated the true number of deaths. To mitigate this, we considered loss to follow‐up as a competing risk for mortality in the analysis. This study was only conducted in Dar es Salaam, the commercial city of Tanzania, and results may not be fully representative of experiences in the entire country, or in other countries in sub‐Saharan Africa. However, to the best of our knowledge, this is first study in Tanzania to assess mortality using routine HIV program data and it involved a large cohort of over 15,000 adolescents and young adults living with HIV. This study provided information on risk factors for mortality for both adolescents and young adults that can be utilized to improve HIV‐related services.

## 5 CONCLUSIONS

The overall mortality rate within 1 year of ART initiation in Dar es Salaam, Tanzania was significantly higher among adolescents than young adults and higher among males than females. Mortality disparity based on age and sex observed in this study warrants the need to explore sex‐ and age‐specific approaches to HIV diagnosis and treatment for adolescents and young adults living with HIV in Tanzania.

## COMPETING INTERESTS

The authors have no competing interests to declare.

## AUTHORS’ CONTRIBUTIONS

MAA, BFS, MMM, FMM, LM and EAA designed the study. MAA, GAS and AR conducted the study, including cleaning the data and preparation of the dataset. MAA, GAS and AR performed the statistical analysis. MAA and GAS wrote the manuscript and all authors contributed to the review and approval of the manuscript.

## FUNDING

Research reported in this publication was supported by the Fogarty International Center of the National Institutes of Health under award number D43TW009775. The content is solely the responsibility of the authors and does not necessarily present the official views of the National Institutes of Health.

## Supporting information


**Table S1**: Comparison of adolescents and young adults’ mortality rates in the first year after ART initiation.

## Data Availability

The data is available upon request.
