# Supplementary material for: Predictors of mortality among adolescents and young adults living with HIV on antiretroviral therapy in Dar es Salaam, Tanzania: a retrospective cohort study
Source: J Int AIDS Soc. 2022 Feb 22;25(2):e25886. doi: 10.1002/jia2.25886 (PMC8863353; doi:10.1002/jia2.25886)
Supplement: Supplementary file 1 — Table S1: Comparison of adolescents and young adults’ mortality rates in the first year after ART initiation. [file JIA2-25-e25886-s001.docx]

Supplemental Table 1: Comparison of adolescents and young adults’ mortality rates in the first year after ART initiation.

| **Age group** | **Dead**  **N (%)** | **Alive**  **N (%)** | **Total**  **N** | **Person Years** | **Mortality Rate [95% CI]** | **Mortality Rate Ratio [95% CI]** |
| --- | --- | --- | --- | --- | --- | --- |
| **Adolescents (10-19 years)** | 114 (2.3%) | 4,847 (97.7%) | 4,961 | 3,005 | 3.8 [3.2-4.6] | 1.8[1.4-2.3] |
| **Young Adults (20-24 years)** | 135 (1.2%) | 10,778 (98.8%) | 10,913 | 6,287 | 2.1 [1.8-2.5] | 1.0 |
|  | **249 (1.6%)** | **15,625 (98.4%)** | **15,874** | 9,292 | **2.7[2.4-3.0]** |  |
